# Lowering Saturated Fat and Increasing Vegetable and Fruit Intake May Increase Insulin Sensitivity 2 Years Later in Children with a Family History of Obesity

**DOI:** 10.1093/jn/nxy189

**Published:** 2018-11-01

**Authors:** Andraea Van Hulst, Gilles Paradis, Soren Harnois-Leblanc, Andrea Benedetti, Vicky Drapeau, Mélanie Henderson

**Affiliations:** 1 Ingram School of Nursing, McGill University, Montreal, Canada; 2 Departments of Epidemiology, Biostatistics, and Occupational Health, McGill University, Montreal, Canada; 3 Medicine, McGill University, Montreal, Canada; 4 Centre Hospitalier Universitaire Sainte-Justine Research Center, Montreal, Canada; 5 School of Public Health, University of Montreal, Montreal, Canada; 6 Faculty of Educational Sciences, Department of Physical Education; 7 Institute of Nutrition and Functional Foods, Laval University, Quebec City, Canada; 8 Quebec Heart and Lung Institute/Research Center, Quebec City, Canada; 9 Division of Endocrinology, Department of Pediatrics, Centre Hospitalier Universitaire Sainte-Justine and University of Montreal, Montreal, Canada

**Keywords:** adiposity, adolescent/children, diet, fat intake, insulin sensitivity, insulin secretion, nutrients, obesity

## Abstract

**Background:**

Identifying dietary factors that determine insulin sensitivity and secretion in children entering puberty may provide valuable information for the early prevention of type 2 diabetes.

**Objectives:**

We assessed whether macronutrients and food groups are longitudinally associated with insulin sensitivity and secretion over a 2-y period in children with a family history of obesity, and whether associations differ by level of adiposity.

**Methods:**

Data were derived from the Quebec Adipose and Lifestyle Investigation in Youth (QUALITY) Study, an ongoing prospective cohort including 630 children recruited at ages 8–10 y, with ≥1 obese parent, and followed 2 y later (*n* = 564). The intake of macronutrients and foods was assessed at baseline using three 24-h dietary recalls. At age 10–12 y, insulin sensitivity was assessed by the Matsuda Insulin Sensitivity Index (ISI) and the homeostatic model assessment of insulin resistance. Insulin secretion was assessed by the ratio of the area under the curve of insulin to the area under the curve of glucose at 30 min and at 120 min of an oral-glucose-tolerance test. Multivariable linear regression models were fitted for each dietary factor while adjusting for age, sex, puberty, physical activity, screen time, total energy intake, and percentage of body fat; and interaction terms between dietary factors and percentage of body fat were tested.

**Results:**

Saturated fat intake was associated with a 1.95% lower (95% CI: −3.74%, −0.16%) Matsuda ISI, whereas vegetable and fruit intake was associated with a 2.35% higher (95% CI: 0.18%, 4.52%) Matsuda ISI 2 y later. The association of saturated fat intake with insulin sensitivity was most deleterious among children with a higher percentage of body fat (*P*-interaction = 0.023). Other than fiber intake, no longitudinal associations between dietary intake and insulin secretion were found.

**Conclusions:**

Lowering saturated fat and increasing vegetable and fruit intakes during childhood may improve insulin sensitivity as children enter puberty. This study was registered at www.clinicaltrials.gov as NCT03356262.

## Introduction

The substantial increases in body weight in North American children and youth have been associated with increases in the prevalence of prediabetic conditions and type 2 diabetes (1–4). Clinical and public health prevention strategies for prediabetes and type 2 diabetes in children and youth are urgently needed. One avenue for prevention may focus on specific dietary factors early on in childhood (5). Dietary factors may increase the risk of type 2 diabetes directly through their impact on glucose-insulin responses and indirectly by promoting excessive weight gain (5).

Studies on the link between dietary intake and glucose-insulin responses in children have focused on dietary factors, such as fat (6, 7), carbohydrate (6, 8), fiber (8–10), or glycemic index (11–13), or specific types of foods characterized as healthy (14). However, few prospective studies have been conducted. In one longitudinal study in 774 adolescent females aged 16–17 y and followed 2 y later, White et al. (8) reported improved insulin sensitivity with higher baseline intake of PUFAs and no effect of MUFA and SFA intake, suggesting that types of fat may be differently associated with insulin dynamics. Similarly, these authors found fiber intake to be prospectively associated with improved insulin sensitivity (8). Other studies have examined relatively short-term effects of dietary interventions on insulin dynamics. For example, a cluster-randomized controlled trial showed that providing Danish children aged 8–11 y with healthy school meals rich in fiber, vegetables, and fish resulted in a small decrease in insulin resistance 3 mo later (14).

In this study, we address limitations of existing studies, namely the need for longitudinal studies that use validated methods to measure dietary intake and insulin dynamics in children and that consider other lifestyle habits that may confound associations. Specifically, we aimed to assess whether habitual dietary intakes of both specific macronutrients and food groups are associated with insulin sensitivity and insulin secretion over a 2-y period in children with a family history of obesity. A secondary objective was to assess whether associations differed by baseline adiposity.

## Methods

### 

Participants were drawn from the Quebec Adipose and Lifestyle Investigation in Youth (QUALITY) Study, an ongoing longitudinal investigation of the natural history of obesity and cardiovascular disease risk factors in white youth. Children were recruited through elementary schools located within 3 major urban centers in Quebec, Canada. Eligibility criteria required participants to be white (Caucasian), aged 8–10 y at recruitment, with both biological parents available to participate in baseline data collection and ≥1 parent being obese on the basis of BMI or abdominal obesity. At baseline, 630 families participated in a clinic visit (2005–2008). A similar follow-up assessment was conducted 2 y later (2007– 2011), when children were aged 10–12 y (*n* = 564) (**Supplemental Figure 1**). Overall, participants lost to follow-up had a higher percentage of body fat mass, lower insulin sensitivity, and higher insulin secretion, and had lower intakes of fiber, grain products, and milk and alternatives compared with those who remained in the study (**Supplemental Table 1**). Written informed consent was obtained from parents, and assent was provided by children. All of the procedures undertaken in this study are in accordance with the standards of the Ethics Review Boards of the CHU Sainte-Justine and the Quebec Heart and Lung Institute, which approved the study. A detailed description of the study design and data collection methods is available (15). The QUALITY Study is registered at www.clinicaltrials.gov (NCT03356262).

#### Dietary assessment

Children's dietary intake was measured at baseline with the use of mean values of three 24-h diet recalls conducted by trained dietitians on nonconsecutive days, including 1 weekend day (16). Except in unusual circumstances, the recalls were collected within a 4-wk period after the baseline clinic visit. Diet recall interviews were conducted by telephone with the child and then confirmed with the parent who prepared the meals. During the clinical visit, each participant was given a small disposable kit containing food-portion models (e.g., graduated cup and bowl) as well as training on the use of the kit during the telephone interview.

Foods reported on the recalls were entered into CANDAT software (Godin and Assoc, London, ON) and converted to nutrients using the 2007 Canadian Nutrient File. Outliers in the analysis of the distribution of each nutrient were examined. Total fiber intake was measured in grams, and total energy intake in kilocalories. Average daily percentages of total energy from dietary fat, SFAs, PUFAs, MUFAs, protein, and carbohydrate were calculated. Daily servings of vegetables and fruit, grain products, milk and alternatives, and meat and alternatives were based on portion sizes from Canada's Food Guide (17).

#### Insulin dynamics

All of the participants underwent a 2-h oral-glucose-tolerance test (OGTT) after a 12-h overnight fast. Blood samples were collected at 30-, 60-, 90-, and 120-min intervals after an oral-glucose dose of 1.75 g/kg body weight (maximum: 75 g). Plasma insulin was measured by using the ultrasensitive Access immunoassay system (Beckman Coulter, Inc.), which has no cross-reactivity with proinsulin or C-peptide (18). Plasma glucose concentrations were computed on the Beckman Coulter Synchron LX20 automat with the use of the glucose oxidase method. Analyses were performed in batches at the Centre Hospitalier Universitaire Sainte-Justine Clinical Biochemistry Laboratory twice monthly. The HOMA-IR, calculated as the product of fasting glucose (millimoles per liter) and fasting insulin (milli-units per liter) divided by 22.5, was used as a measure of fasting insulin sensitivity (19). In addition, the OGTT-derived Matsuda insulin sensitivity index (Matsuda ISI) was computed as 10,000/[(fasting glucose × fasting insulin) × (mean OGTT glucose × mean OGTT insulin)] (20). Both the HOMA-IR and the Matsuda ISI have previously been validated in children aged 6–18 y (21). Insulin secretion was measured by using OGTT-derived measures, namely the ratio of the AUC of insulin to the AUC of glucose during the first 30 min (AUC I/G_t30min_) of the OGTT (first-phase insulin secretion) and during the full 2 h (AUC I/G_t120min_) of the OGTT (second-phase insulin secretion). The AUC I/G_t30min_ has previously been found to be an accurate estimate of first-phase insulin secretion in healthy children (22).

#### Other measurements

Physical activity was assessed with the use of 7-d accelerometry (Actigraph LS 7164 activity monitor; Actigraph LLC). Valid wear time consisted of a minimum of 10 h/d for a minimum of 4 d. Nonwear time was defined as any period of ≥60 min of 0 counts, accepting 1 min or 2 consecutive minutes where count values were >0 and ≤100 (23). Moderate-to-vigorous physical activity was computed by adding the total minutes spent daily on moderate and vigorous physical activities and averaging over the total number of valid days of wear (24, 25). Screen time was assessed by using an interviewer-administered questionnaire to document self-reported habitual daily hours spent on leisure television viewing and computer/video game use on weekdays and weekends. The average daily hours of leisure screen time was calculated. Body composition was measured by DXA. Percentage body fat mass was calculated as total fat mass/total body mass × 100. Pubertal development stage was assessed by a trained nurse with the use of the 5-stage Tanner scale (26, 27), and was dichotomized as prepubertal (Tanner 1) or puberty initiated (Tanner >1).

#### Statistical analysis

Descriptive statistics were used to characterize participants at baseline and follow-up. Spearman's rank-order correlation coefficients were computed for nonnormally distributed continuous variables measured at both time points. Multivariable linear regression analyses were used to examine the association of each dietary factor with Matsuda ISI, HOMA-IR, and insulin secretion (AUC I/G_t30min_ and AUC I/G_t120min_) in distinct models while adjusting for sex, age, and pubertal development at follow-up; mean minutes of moderate-to-vigorous physical activity; hours of screen time per day; and total energy intake as well as percentage body fat mass at baseline. Insulin sensitivity and secretion variables were transformed (100 × ln of variable) to normalize their distribution. The interpretation of β coefficients is therefore as follows: for a 1-unit increase in the independent variable, β represents the percentage increase (for positive β) or decrease (for negative β) in the outcome (28). Models for insulin secretion were also adjusted for insulin sensitivity using fractional polynomials to account for their established nonlinear association (29). All associations were tested for nonlinearity with the use of nonparametric smoothing splines. These findings are not presented given that no nonlinear associations were found between dietary factors and insulin outcomes. Interactions between each macronutrient or food group and percentage body fat mass were tested by including interaction terms to covariable-adjusted models.

Sensitivity analyses were conducted. First, we repeated regression analyses on 20 imputed data sets created by using multiple imputations with the fully conditional specification. Second, to examine associations between diet and 2-y changes in insulin sensitivity (Matsuda ISI and HOMA-IR), the model for Matsuda ISI at follow-up was adjusted for baseline Matsuda ISI, and the model for HOMA-IR at follow-up was adjusted for baseline HOMA-IR. Third, we tested associations for the intake of saturated fat from specific sources of foods, namely from dairy sources only and from meat sources only, given recent findings suggesting that these may be differently associated with the risk of type 2 diabetes in adult populations (30, 31). All of the analyses were conducted with SAS version 9.4 (SAS Institute).

## Results

Characteristics of the 564 QUALITY participants who completed both baseline and follow-up visits are presented in **Table 1**. At follow-up, 17% were overweight, 22% were obese, and the majority had initiated puberty (67%). Over the 2-y follow-up period, percentage body fat mass increased by 2.3%. As expected given the pubertal stages of participants, insulin sensitivity decreased and first- and second-phase insulin secretion increased between baseline and follow-up assessments.

**TABLE 1:** Characteristics of participants who completed the baseline and follow-up visit of the QUALITY study^1^

	Baseline (time 1)	Follow-up (time 2)	Correlation between time 1 to time 2 (*r*_s_)
Age, y	9.6 ± 0.9	11.7 (0.9)	
Male sex	55.5	—	
BMI category			
Normal weight	58.9	60.3	
Overweight	19.5	17.4	
Obese	21.6	22.3	
Pubertal (Tanner stage >1)	20.6	67.0	
Percentage body fat, %	26.1 ± 10.8	28.4 ± 10.9	0.9
MVPA, min/d	47.7 (31.1–64.7)	43.1 (26.6–55.9)	0.6
Screen time, h/d	2.2 (1.3–3.6)	2.9 (1.9–4.4)	0.5
HOMA-IR	0.8 (0.6–1.2)	1.2 (0.8–1.9)	0.6
Matsuda ISI	9.4 (6.3–12.9)	6.6 (4.4–9.6)	0.7
AUC I/G			
30 min	25.9 (17.6–39.8)	35.7 (24.2–53.5)	0.6
120 min	26.9 (19.7–39.8)	36.3 (24.8–55.4)	0.6
Total energy intake, kcal/d	1702 ± 392	—	
Carbohydrate, %	53.0 ± 6.2	—	
Total fat, %	32.3 ± 4.9	—	
SFAs, %	11.5 ± 2.6	—	
PUFAs, %	5.5 ± 1.8	—	
MUFAs, %	11.3 ± 2.4	—	
Protein, %	16.0 ± 3.2	—	
Fiber, g/d	13.4 ± 4.2	—	
Vegetables and fruit, servings/d	4.4 ± 2.1	—	
Grain products, servings/d	4.7 ± 1.7	—	
Meat and alternatives, servings/d	1.9 ± 0.8	—	
Milk and alternatives, servings/d	1.9 ± 1.0	—	

^1^Values are percentages, medians (IQRs), or means ± SDs; *n* = 564. Spearman's ρ between selected variables measured at time 1 and time 2 are shown. AUC I/G, area under the curve of insulin to the area under the curve of glucose; ISI, insulin sensitivity index; MVPA, moderate-to-vigorous physical activity; QUALITY, Quebec Adipose and Lifestyle Investigation in Youth.

Main effect associations between baseline dietary intake and the Matsuda ISI and HOMA-IR are presented in **Table 2**. Every incremental increase in SFAs as a percentage of total energy intake at baseline was associated with a Matsuda ISI that was lower by 1.95% (95% CI: −3.74%, −0.16%) 2 y later. In contrast, PUFAs and MUFAs were not associated with insulin sensitivity. In sensitivity analyses, when examining dietary sources of saturated fat, we observed that this association held for SFAs from dairy sources, but not for SFAs from meat sources (**Supplemental Table 2**). Every additional serving of vegetables and fruit at baseline was associated with a 2.35% (95% CI: 0.18%, 4.52%) higher Matsuda ISI 2 y later. In sensitivity analyses, SFAs (*P* = 0.06) and vegetable and fruit intake (*P* = 0.07) remained marginally associated with 2-y changes in the Matsuda ISI (**Supplemental Table 3**). However, when considering both SFAs and vegetable and fruit intake in a single model, associations with the Matsuda ISI were attenuated toward the null (data not shown). When using HOMA-IR as a measure of fasting insulin sensitivity, associations with SFAs and vegetables and fruit servings were generally similar in effect size, albeit without reaching significance. Results were similar between case-complete and imputed data analysis, although with a loss of significance (Table 2). Moreover, fiber intake was associated with improved Matsuda ISI (1.33%; 95% CI: 0.18%, 2,47%) and was borderline associated with improved HOMA-IR (−1.10%; 95% CI: −2.27%, 0.07%) in imputed analyses only.

**TABLE 2:** Associations between dietary factors at age 8–10 y and insulin sensitivity (Matsuda ISI) and insulin resistance (HOMA-IR) 2 y later: QUALITY cohort^1^

	Matsuda ISI		HOMA-IR	
	Case complete (*n* = 443)	Imputed (*n* = 534)	Case complete (*n* = 454)	Imputed (*n* = 548)
Carbohydrates, %	0.56 (−0.15, 1.27)	0.52 (−0.13, 1.17)	−0.65 (−1.38, 0.07)	−0.61 (−1.27, 0.05)
Total fat, %	−0.77 (−1.70, 0.15)	−0.65 (−1.48, 0.17)	0.75 (−0.20, 1.71)	0.70 (−0.15, 1.55)
SFAs, %	−1.95 (−3.74, −0.16)*	−1.31 (−2.88, 0.25)	1.40 (−0.45, 3.26)	0.84 (−0.75, 2.42)
PUFAs, %	−0.86 (−3.34, 1.62)	−0.98 (−3.22, 1.26)	1.03 (−1.51, 3.57)	1.60 (−0.66, 3.86)
MUFAs, %	−0.24 (−2.15, 1.68)	−0.52 (−2.23, 1.19)	0.82 (−1.15, 2.79)	0.92 (−0.81, 2.66)
Protein, %	−0.007 (−1.38, 1.37)	−0.05 (−1.31, 1.22)	0.27 (−1.15, 1.70)	0.15 (−1.14, 1.45)
Fiber, g/d	1.02 (−0.27, 2.32)	1.33 (0.18, 2.47)*	−0.98 (−2.32, 0.37)	−1.10 (−2.27, 0.07)
Vegetables and fruit, servings/d	2.35 (0.18, 4.52)*	2.24 (0.25, 4.24)*	−2.18 (−4.43, 0.06)	−2.06 (−4.10, −0.03)*
Grain products, servings/d	−0.06 (−3.40, 3.28)	0.66 (−2.28, 3.61)	0.72 (−2.69, 4.13)	−0.02 (−2.99, 2.94)
Meat and alternatives, servings/d	−1.42 (−7.30, 4.46)	−1.64 (−6.99, 3.71)	2.79 (−3.33, 8.91)	2.61 (−2.90, 8.12)
Milk and alternatives, servings/d	−0.62 (−5.74, 4.50)	0.29 (−4.24, 4.83)	−0.64 (−5.91, 4.64)	−1.88 (−6.51, 2.76)

^1^Values are β coefficients (95% CIs). For every incremental increase in saturated fat as a percentage of total energy intake at age 8–10 y, the Matsuda ISI is lower by 1.95% at age 10–12 y. Models were adjusted for exact age at follow-up, sex, Tanner stage at follow-up, MVPA, screen time, total energy intake, and adiposity at baseline. *P < 0.05. ISI, insulin sensitivity index; MVPA, moderate-to-vigorous physical activity; QUALITY, Quebec Adipose and Lifestyle Investigation in Youth.

First-phase insulin secretion was not associated with any of the dietary factors examined nor was second-phase insulin secretion, except for fiber intake (**Table 3**). Every additional daily gram of fiber intake at baseline was associated with a 0.83% (95% CI: 0.01%, 1.65%) higher second-phase insulin secretion 2 y later, but only the in case-complete analyses.

**TABLE 3:** Associations between dietary factors at age 8–10 y and first-phase (AUC I/G 30 min) and second-phase insulin secretion (AUC I/G 120 min) 2 y later: QUALITY cohort^1^

	AUC I/G			
	30 min		120 min	
	Case complete (*n* = 443)	Imputed (*n* = 534)	Case complete (*n* = 443)	Imputed (*n* = 534)
Carbohydrates, %	0.03 (−0.52, 0.58)	0.07 (−0.44, 0.57)	0.19 (−0.26, 0.64)	0.14 (−0.28, 0.55)
Total fat, %	0.20 (−0.51, 0.92)	0.09 (−0.55, 0.72)	−0.30 (−0.88, 0.29)	−0.22 (−0.75, 0.31)
SFAs, %	−0.04 (−1.43, 1.36)	−0.05 (−1.25, 1.15)	−0.21 (−1.35, 0.92)	−0.09 (−1.09, 0.90)
PUFAs, %	1.35 (−0.56, 3.26)	0.97 (−0.75, 2.69)	−0.35 (−1.91, 1.21)	−0.65 (−2.08, 0.79)
MUFAs, %	0.84 (−0.64, 2.31)	0.70 (−0.61, 2.02)	−0.92 (−2.13, 0.28)	−0.67 (−1.76, 0.42)
Protein, %	−0.46 (−1.52, 0.60)	−0.40 (−1.37, 0.58)	0.14 (−0.72, 1.01)	0.17 (−0.63, 0.96)
Fiber, g/d	0.06 (−0.95, 1.06)	−0.10 (−0.99, 0.79)	0.83 (0.01, 1.65)*	0.57 (−0.16, 1.30)
Vegetables and fruit, servings/d	−0.21 (−1.90 1.48)	−0.29 (−1.84, 1.26)	0.65 (−0.73, 2.03)	0.49 (−0.78, 1.77)
Grain products, servings/d	1.31 (−1.26, 3.89)	0.95 (−1.31, 3.20)	−0.49 (−2.60, 1.61)	−0.75 (−2.63, 1.12)
Meat and alternatives, servings/d	−1.30 (−5.84, 3.24)	−0.70 (−4.87, 3.47)	−1.10 (−4.81, 2.61)	−0.11 (−3.52, 3.31)
Milk and alternatives, servings/d	−2.68 (−6.62, 1.27)	−2.59 (−6.09, 0.90)	1.54 (−1.68, 4.77)	1.09 (−1.82, 3.99)

^1^Values are β coefficients (95% CIs). For every incremental increase in the intake of grams of fiber per day at age 8–10 y, second-phase insulin secretion (AUC I/G_120min_) is higher by 0.83% at age 10–12 y. Models were adjusted for exact age at follow-up, sex, Tanner stage at follow-up, MVPA, screen time, total energy intake, adiposity at baseline, and insulin sensitivity. *P < 0.05. AUC I/G, AUC of insulin to the AUC of glucose; MVPA, moderate-to-vigorous physical activity; QUALITY, Quebec Adipose and Lifestyle Investigation in Youth.

Interactions were found between baseline saturated fat intake and percentage body fat mass (*P*-interaction = 0.023), and between baseline portions of grain products and percentage body fat mass (*P*-interaction = 0.006) in relation to the Matsuda ISI. When stratifying associations by tertiles of percentage body fat mass, we found a negative association between SFAs intake and Matsuda ISI only among children within the highest tertile of percentage body fat mass (β = −4.29%; 95% CI: −7.98%, −0.61%), and no associations for those in other tertiles of adiposity (**Table 4**). Similarly, among children within the lowest tertile of percentage body fat mass, the number of daily servings of grain products was positively associated with the Matsuda ISI (β = 5.10%, 95% CI: −0.16%, 10.35%), whereas it was negatively associated in the highest tertile of percentage body fat mass (β = −4.07%, 95% CI: −10.85%, 2.71%). Comparable interactions were found for HOMA-IR.

**TABLE 4:** Associations between selected dietary factors at age 8–10 y and insulin sensitivity (Matsuda ISI) 2 y later by baseline level of adiposity (QUALITY cohort)^1^

	β (95% CI)		
	Lowest tertile of % body fat (*n* = 148)	Middle tertile of % body fat (*n* = 151)	Highest tertile of % body fat (*n* = 144)
SFAs, %	−0.78 (−3.86, 2.29)	−0.001 (−3.04, 3.03)	−4.29 (−7.98, −0.61)
Grain products, servings/d	5.10 (−0.16, 10.35)	−0.68 (−6.80, 5.45)	−4.07 (−10.85, 2.71)

^1^Models are adjusted for age, sex, and Tanner stage at follow-up, and for total energy intake, physical activity, and screen time at baseline. ISI, insulin sensitivity index; QUALITY, Quebec Adipose and Lifestyle Investigation in Youth.

## Discussion

Children whose usual diet contains a higher percentage of energy from SFAs and fewer servings of vegetables and fruit had lower insulin sensitivity 2 y later; these associations were independent of other lifestyle habits and of adiposity, and even of baseline insulin sensitivity. The deleterious effect of SFAs intake increased with higher baseline adiposity. Similarly, the number of servings of grain products was associated with lower insulin sensitivity among those with higher baseline adiposity; this association was, however, inverse among children who were lean at baseline. We found little evidence for associations between dietary factors and insulin secretory demands. Findings from this study should be interpreted with caution given the number of associations tested and the increased risk of type 1 error. Results should thus be seen as exploratory and need to be confirmed in future studies.

The potential contribution of dietary fat on type 2 diabetes has been the subject of many studies in adult populations. In their systematic review and meta-analysis of 102 trials, Imamura et al. (32) found that replacing dietary saturated fat with mono- or polyunsaturated fat improved glucose-insulin homeostasis. The literature in pediatric populations is much scarcer, particularly with respect to prospective studies. As in the adult literature, it has been shown that different types of dietary fat may have distinct effects on later insulin sensitivity in children and adolescents (8, 14, 33). We observed a deleterious effect of baseline SFAs intake but no associations for PUFA or MUFA intake, unlike White et al. (8), who reported improved insulin sensitivity with higher PUFA intake. A recent prospective study in Canadian children similar in age to those in the QUALITY study reported no association between baseline total fat intake and insulin sensitivity after adjustment for confounders; however, associations for specific types of fat were not reported (34).

Underlying mechanisms for associations between fat intake and insulin resistance have been proposed (35, 36). In adults and in animal studies, diet-induced inflammation, via various dietary FAs such as n–6 FAs and SFAs, has been associated with the development of insulin resistance (35). More recently, studies have pointed to the potential contribution of fat, specifically from dairy sources, to reduce insulin resistance and type 2 diabetes in adults (37–39), with limited evidence to date in children (40, 41). In sensitivity analyses, we observed the opposite: SFAs from dairy sources, particularly when including dairy foods with added sugar, were associated with lower insulin sensitivity. In contrast, no association was observed for SFAs from meat sources. Further studies in pediatric populations are needed to better understand the potential impact of SFAs from different dietary sources on type 2 diabetes risk factors in children.

The deleterious effect of SFAs intake on insulin sensitivity increased among participants with higher baseline adiposity. This accords with the inflammation pathway proposed previously: both adiposity and chronic intake of SFAs are associated with inflammatory states, which may, in turn, lead to insulin resistance (35, 42). The stronger association between SFAs and insulin sensitivity in children with higher adiposity may reflect already present cellular inflammation among obese subjects and suggests an additive effect of saturated fat intake and adiposity on insulin sensitivity starting in childhood. With regard to grain products, their intake was associated with higher insulin sensitivity in leaner children and with lower insulin sensitivity in children with more fat mass. One explanation for this finding is that the quality of grain products consumed by lean and obese children may differ, notably in terms of glycemic index and total and type of fiber. A positive association between water-soluble fiber intake and insulin sensitivity has been previously reported (8). We found an association between total fiber intake and insulin sensitivity, but only in imputed data analysis; however, we did not break down grain products on the basis of their glycemic index or type of fiber content.

Vegetable and fruit intake has been inconsistently associated with insulin sensitivity in children (34, 43). Discordant findings between studies may relate to differences in age groups studied, in methods used to measure vegetable and fruit intake and insulin sensitivity, or in residual confounding. In our study, every additional serving of vegetables and fruit increased the Matsuda ISI and decreased HOMA-IR by ∼2% after 2 y. Strategies aimed at increasing access to and intake of vegetables and fruits may lead to improved insulin sensitivity over time and contribute to the prevention of type 2 diabetes.

Overall, associations with HOMA-IR were attenuated compared with those with the Matsuda ISI. The latter relates to total body (i.e., muscle and adipose tissue) insulin sensitivity in a dynamic way after an oral-glucose load, whereas HOMA-IR is a fasting measure related to hepatic insulin sensitivity. Our findings suggest that insulin dynamics in the postprandial state may be more strongly influenced by usual dietary intake compared with hepatic insulin sensitivity measured in the fasting state.

Other than for fiber intake, no longitudinal associations between dietary intake and insulin secretion were found. Our finding of increased second-phase insulin secretory requirements with fiber intake was unexpected. Indeed, although fiber slows the absorption of glucose within a given meal, it is unclear why a higher intake of fiber would alter second-phase insulin secretion in response to an acute glucose load. The absence of any other association between baseline dietary factors and insulin secretion may reflect metabolic plasticity in children (7, 44). It may be that children at a young age compensate for dietary intake by upregulating appropriate metabolic pathways; however, the cumulative effect of dietary intake on glucose-insulin responses from childhood to adolescence to adulthood may result in an adverse impact on insulin secretion over time. Longer follow-up studies are needed to better understand which dietary factors to target for the maintenance of optimal insulin secretory function.

Overall, our findings were similar in terms of magnitude when using either case-complete or imputed data, although with associations attenuated to the null, except in the case of fiber intake, which became associated with the Matsuda ISI. This may relate to the fact that dietary intake is difficult to measure accurately and that multiple imputations on data that are measured with some degree of nondifferential misclassification error could lead to further imprecision in measurements and bias associations toward the null (45).

This study has several strengths, namely its prospective design, the large sample size, and the use of validated methods to measure habitual diet (16) and insulin dynamics (21, 22). We took into consideration several potential confounders, namely physical activity, sedentary behavior, and adiposity. Nevertheless, residual confounding cannot be entirely ruled out in this observational study. Another limitation is the possibility of selection bias given that children lost to follow-up were more likely to be obese, to have lower insulin sensitivity, and to have diets lower in fiber content, grain products, and milk and alternatives. However, this likely would have resulted in an underestimation of associations. Dietary intake was measured at baseline only. Although there is some evidence suggesting that dietary patterns are stable over time during childhood (46), observed associations may be the result of changes in diet over the course of follow-up. Last, findings from our study are generalizable to white children with a parental history of obesity; this group comprises a significant segment of the Canadian population. Overall, the dietary intake of QUALITY participants is comparable to population estimates for children of similar ages, except for servings of grain products, of which they consumed less (47).

In conclusion, this study suggests that, among children with a parental history of obesity, lower SFAs and higher vegetable and fruit intakes are associated with better insulin sensitivity as these children enter puberty. A diet low in SFAs appears to be particularly important for children who already have higher adiposity. Further studies are needed to better understand what underlies differences in the association between the intake of grain products and insulin sensitivity according to adiposity, as well as the impact of SFAs from different dietary sources. Promoting healthy dietary choices, namely increasing intakes of vegetables and fruit and decreasing intakes of SFAs early on in at-risk children, may contribute to preventing the later development of type 2 diabetes.

## Supplementary Material

nxy189_Supplemental_Figure_Tables

## Notes

The Quebec Adipose and Lifestyle Investigation in Youth (QUALITY) cohort is supported by the Canadian Institutes of Health Research (OHF-69442, NMD-94067, MOP-97853, MOP-119512), the Heart and Stroke Foundation of Canada (PG-040291), and the Fonds de la Recherche du Québec–Santé. AVH holds a Canadian Institutes of Health Research Postdoctoral Fellowship and a Fellowship in Preventive Cardiology (funded by the International Conference on Preventive Cardiology/Heart and Stroke Foundation Canada/Canadian Cardiovascular Society). VD is the recipient of the 2015 Yogurt in Nutrition Initiative grant and has received grants from Danone and Dairy Farmers of Canada. MH holds a Diabetes Junior Investigator Award from the Canadian Society of Endocrinology and Metabolism–AstraZeneca and a Fonds de Recherche du Québec–Santé Junior 2 salary award. SHL holds a Doctoral Award from the Fonds de Recherche du Québer-Santé.

Author disclosures: AVH, GP, SHL, AB, VD, and MH, no conflicts of interest. The funders played no role in the design or conduct of the study; collection, management, analysis, or interpretation of the data; the preparation, review, or approval of the manuscript; or the decision to submit the manuscript for publication.

Supplemental Figure 1 and Supplemental Tables 1–3 are available from the “Supplementary data” link in the online posting of the article and from the same link in the online table of contents at https://academic.oup.com/jn/.

Abbreviations used: ISI, insulin sensitivity index; OGTT, oral glucose tolerance test; QUALITY, Quebec Adipose and Lifestyle Investigation in Youth.
