# Supplementary material for: Lowering Saturated Fat and Increasing Vegetable and Fruit Intake May Increase Insulin Sensitivity 2 Years Later in Children with a Family History of Obesity
Source: J Nutr. 2018 Nov 1;148(11):1838–44. doi: 10.1093/jn/nxy189 (PMC6533243; doi:10.1093/jn/nxy189)
Supplement: nxy189_Supplemental_Figure_Tables [file nxy189_supplemental_figure_tables.docx]

**Lowering saturated fat and increasing vegetable and fruit intake may improve insulin sensitivity** **in children with a family history of obesity**

**Supplemental Figure 1.** Recruitment and participation in the QUALITY Cohort

**Families who completed Visit 2: 564**

**(90% retention)**

3350 families contacted to assess their eligibility

387 377 pamphlets distributed in 1040 primary schools (89% of schools approached accepted to distribute the pamphlets)

**1320 families met the eligibility criteria**

- Not eligible: 2030 (61%)

**Families seen for Visit 1: 634 families (48% of eligible families)**

- Chose not to participate: 686 (52%)

**Families invited for Visit 2: 630**

- Refused participation to Visit 2: 41
- Lost to follow-up: 19
- Removed from study by research team: 4 (0.6%)*

* Families were removed from study because child or parents was unable or refused to complete most or all of data collection for baseline assessment after having provided consent to participate

**Supplemental Table 1.** Comparison of baseline characteristics for participants who completed the follow-up visit (n=564) with those who did not (n=66), for a total of 630 participants to the QUALITY study

|  | **Completed follow-up**  **(n=564)** | **Lost to follow-up**  **(n=66)** | **p-value** |
| --- | --- | --- | --- |
| Age (y) | 9.6 ± 0.9 | 9.6 ± 0.9 | 0.326 |
| Sex |  |  | 0.121 |
| Male | 55.5 | 45.5 |  |
| Female | 44.5 | 54.5 |  |
| Parent education |  |  | 0.060 |
| 2 parents with high school degree or less | 7.1 | 13.9 |  |
| At least 1 parent with technical/vocational/trade school degree | 37.2 | 43.1 |  |
| At least 1 parent with university degree | 55.7 | 43.1 |  |
| BMI z-score | 0.7 ± 1.1 | 0.9 ± 1.2 | 0.177 |
| Tanner stage > 1 | 20.6 | 29.2 | 0.107 |
| Percent body fat mass (%) | 26.1 ± 10.8 | 30.0 ± 11.7 | **0.006** |
| MVPA (min/d) | 47.7 (31.1 – 64.7) | 47.1 (31.9 – 63.7) | 0.938 |
| Screen time (hr/d) | 2.2 (1.3 – 3.6) | 3.2 (1.4 – 4.3) | **0.045** |
| Matsuda-ISI | 9.4 (6.3 – 12.9) | 8.6 (4.4 – 11.3) | **0.008** |
| HOMA-IR | 0.8 (0.6 – 1.2) | 1.1 (0.7 – 1.7) | **0.006** |
| AUC I/G 30 min | 25.9 (17.6 – 39.8) | 31.4 (20.1 – 55.4) | **0.020** |
| AUC I/G 120 min | 26.9 (19.7 – 39.8) | 30.7 (19.5 – 57.3) | 0.051 |
| Total energy intake (kcal/d) | 1702 ± 392 | 1555 ± 392 | **0.006** |
| Carbohydrate (%) | 53.0 ± 6.2 | 51.9 ± 7.2 | 0.197 |
| Total fat (%) | 32.3 ± 4.9 | 32.1 ± 4.6 | 0.766 |
| Saturated fat (%) | 11.5 ± 2.6 | 11.2 ± 2.3 | 0.324 |
| PUFA (%) | 5.5 ± 1.8 | 5.3 ± 1.7 | 0.309 |
| MUFA (%) | 11.3 ± 2.4 | 11.3 ± 2.6 | 0.875 |
| Protein (%) | 16.0 ± 3.2 | 17.2 ± 4.2 | **0.050** |
| Fiber (g/d) | 13.4 ± 4.2 | 12.1 ± 4.1 | **0.026** |
| Vegetables and fruit (serving/d) | 4.4 ± 2.1 | 4.0 ± 2.1 | 0.254 |
| Grain products (serving/d) | 4.7 ± 1.7 | 4.3 ± 1.6 | **0.042** |
| Meat and alternatives (serving/d) | 1.9 ± 0.8 | 1.9 ± 1.0 | 0.991 |
| Milk and alternatives (serving/d) | 1.9 ± 1.0 | 1.6 ± 0.9 | **0.046** |

Values are percentages, medians (IQRs), or means ± SDs. P-values are for chi-squared tests in the case of categorical variables and for t-tests (or Mann-Whitney tests) when comparing continuous variables.

BMI, body mass index; MVPA, moderate-to-vigorous physical activity; AUC I/G, area under the curve of insulin to the area under the curve glucose; IQR, inter-quartile range.

**Supplemental Table 2.** Prospective associations between saturated fat intake at 8-10 years by food source and insulin sensitivity (Matsuda ISI) and insulin resistance (HOMA-IR) 2 years later in the QUALITY cohort

|  | Matsuda ISI  n=443 | | HOMA-IR  n=454 | |
| --- | --- | --- | --- | --- |
|  | Beta (95% CI) | | | |
| Saturated fat from dairy products (%) | -2.61 | (-4.71, -0.51)^†^ | 1.89 | (-0.27, 4.05) ^‡^ |
| Saturated fat from dairy products with no added sugar (%) | -2.02 | (-4.40, 0.35) ^‡^ | 1.61 | (-0.82, 4.05) |
| Saturated fat from meat products (%) | -0.93 | (-4.01, 2.15) | 0.56 | (-2.60, 3.72) |

Analysis are for case-complete data. Models are adjusted for exact age at follow-up, sex, Tanner stage at follow-up, MVPA, screen time, total energy intake and adiposity at baseline; † indicates p-value <0.05; ‡ indicates p-value <0.10

**Supplemental Table 3.** Associations between dietary factors at 8-10 years and *2-year changes* in insulin sensitivity (Matsuda ISI) and insulin resistance (HOMA-IR) in the QUALITY cohort

|  | Matsuda ISI  n=419 | | HOMA-IR  n=452 | |
| --- | --- | --- | --- | --- |
|  | Beta (95% CI) | | | |
| Carbohydrates (%) | 0.20 | (-0.45, 0.86) | -0.48 | (-1.17, 0.21) |
| Fat (%) | -0.48 | (-1.33, 0.37) | 0.59 | (-0.31, 1.49) |
| Saturated fat (%) | -1.60 | (-3.25, 0.05) ^‡^ | 1.01 | (-0.74, 2.75) |
| PUFA (%) | -0.93 | (-3.21, 1.36) | 1.29 | (-1.09, 3.68) |
| MUFA (%) | 0.54 | (-1.25, 2.31) | 0.43 | (-1.43, 2.28) |
| Protein (%) | 0.44 | (-0.81, 1.70) | 0.02 | (-1.32, 1.37) |
| Fiber (g/d) | 0.59 | (-0.63, 1.82) | -0.60 | (-1.87, 0.66) |
| Vegetables and fruit (serving/d) | 1.93 | (-0.13, 3.99) ^‡^ | -1.90 | (-4.01, 0.21) |
| Grain products (serving/d) | -0.35 | (-3.42, 2.72) | 0.84 | (-2.35, 4.03) |
| Meat and alternatives (serving/d) | 1.96 | (-3.54, 7.46) | 0.47 | (-5.30, 6.25) |
| Milk and alternatives (serving/d) | -0.10 | (-4.81, 4.61) | -0.72 | (-5.66, 4.23) |

Analysis are for case-complete data. Models are adjusted for exact age at follow-up, sex, Tanner stage at follow-up, MVPA, screen time, total energy intake and adiposity at baseline, as well as for outcome variables measured at baseline. ‡ indicates p-value <0.10
